# Integrin Signaling in the Central Nervous System in Animals and Human Brain Diseases

**DOI:** 10.3390/ijms23031435

**Published:** 2022-01-27

**Authors:** Hiroko Ikeshima-Kataoka, Chikatoshi Sugimoto, Tatsuya Tsubokawa

**Affiliations:** 1 Department of Biology, Keio University, 4-1-1, Hiyoshi, Kohoku-ku, Yokohama-shi 223-8521, Japan; chikatoshi.sugimoto@keio.jp (C.S.); tsubo@a8.keio.jp (T.T.); 2 Faculty of Science and Engineering, Waseda University, 3-4-1 Okubo, Shinjuku-ku, Tokyo 169-8555, Japan

**Keywords:** astrocyte, axon, CNS, invertebrate, mammal, mouse, neuron, phylogenetic tree, regeneration, teleost fish

## Abstract

The integrin family is involved in various biological functions, including cell proliferation, differentiation and migration, and also in the pathogenesis of disease. Integrins are multifunctional receptors that exist as heterodimers composed of α and β subunits and bind to various ligands, including extracellular matrix (ECM) proteins; they are found in many animals, not only vertebrates (e.g., mouse, rat, and teleost fish), but also invertebrates (e.g., planarian flatworm, fruit fly, nematodes, and cephalopods), which are used for research on genetics and social behaviors or as models for human diseases. In the present paper, we describe the results of a phylogenetic tree analysis of the integrin family among these species. We summarize integrin signaling in teleost fish, which serves as an excellent model for the study of regenerative systems and possesses the ability for replacing missing tissues, especially in the central nervous system, which has not been demonstrated in mammals. In addition, functions of astrocytes and reactive astrocytes, which contain neuroprotective subpopulations that act in concert with the ECM proteins tenascin C and osteopontin via integrin are also reviewed. Drug development research using integrin as a therapeutic target could result in breakthroughs for the treatment of neurodegenerative diseases and brain injury in mammals.

## 1. Introduction

Integrins are multifunctional receptors mainly responsible for binding to extracellular matrix (ECM) proteins that mediate cell adhesion. Cell–cell and cell–ligand adhesions were observed and determined to be important processes based in tissue culture studies conducted by Harrison in the early 20th century [1]. Tissue culture techniques came to be widely used for cancer research in the 1960s and early 1970s. In one study reported, in 1976, that when fibronectin, among the earliest of the identified ECM proteins, was added to a culture of viral-transformed cells, the cells reverted to their normal phenotype. In 1978, receptors for fibronectin were biochemically purified as a 140 kD protein complex and immunologically identified by monoclonal antibodies, and were named integrins, to reflect their integral membrane-protein nature and their role in linking the cytoskeleton to the ECM (fibronectin) [2,3]. Then, in 1986, the cDNA of the β integrin subunit was cloned. Bioassays have revealed that the Arg-Gly-Asp-Ser (RGDS) motif is sufficient to inhibit the binding of fibronectin to integrins [4]. Monoclonal antibodies directed against lymphocytes have been shown to share a common subunit and adhesion-related processes with leucocyte-function-associated antigen (LFA1) and macrophage 1 antigen (MAC1). The *Drosophila melanogaster* position-specific antigen (PS) was characterized as a heterodimeric complex that shared a common subunit. All of these findings were reported between 1986 and 1987 [5,6,7].

In regard to their molecular structure, integrins are composed of a large extracellular domain that binds to the ECM and other proteins, a transmembrane domain, and a short intercellular domain that interacts with signal transduction proteins composed of about 20–60 amino acids. However, the cytoplasmic domain of integrin β4 is unusually large (about 1000 a.a.) that interact with cytoskeletal components of hemidesmosomes [8]. The fully functional integrins exist as heterodimers composed of one α integrin subunit and one β integrin subunit. Currently, mammals have been reported to have 18 different α subunits and 8 β subunits of integrin that form 24 α/β pairs in a specific way [9]. Some pairs (α1β1, etc.) are known as collagen receptors, others (αDβ2, etc.) as leucocyte integrins, and some others (α9β1, etc.) as osteopontin receptors. In the regeneration of the peripheral nervous system (PNS), ID1/3 transcription factors regulate the expression of α6 integrin, which has been shown to mediate interactions between axons and skin fibroblasts to promote axon extension [10]. In central nervous system (CNS) regeneration, fibrotic scars formed by fibroblasts may exert inhibitory effects both via the production of inhibitory molecules (CSPG, collagen, etc.) and by physical abrogation of aligned pathways for regenerating axons crossing the lesion [11].

In mammals, some of the integrin receptors are involved in peripheral nerve regeneration [12,13]. The localization of the integrin family in the adult nervous system and the roles of integrins in axonal regeneration have been elegantly summarized in some review articles [14,15]. Integrin function in concert with ECM in synaptic plasticity and memory are important in the brain, and the roles of integrins in brain diseases have been summarized [16].

Integrins have various roles in neuronal connectivity and development of the brain [17,18]. In the adult mammalian CNS, injured axons fail to regenerate; while they have the capacity to regenerate during development, this ability is lost with maturity. Many researchers have focused on the molecules that contribute to CNS development to induce regeneration of the CNS after injury, however, good results have not yet been obtained. For example, the axonal growth-promoting integrins continue to be transported until adulthood in the axons of the PNS, while such transport declines with maturity in axons of the CNS. In fact, integrin and Rab11, a regulator of recycling endosomes, manipulated into mature CNS axons in vitro, enabled axonal regeneration after laser injury, but long-distance regeneration in vivo remains to be observed [13].

In the mammalian CNS, astrocytes are among the most abundant of glial cells that maintain the physiological integrity of the blood brain barrier (BBB), and play roles in glucose sensing (so-called astrocyte-neuron lactate shuttle hypothesis), blood flow regulation, synaptic functioning at tripartite synapses, gliotransmission, etc. [19,20,21]. Recently, we summarized the metabolic plasticity of astrocytes and aging of the brain in a review article [22]. The distribution and expressions of three astrocytic markers, namely, glial fibrillary acidic protein (GFAP), Ca^2+^ binding peptide (S100β), and N-Myc downstream-regulated gene 2 (NDRG2), in different mouse cerebral regions has been reported [23]. Since astrocytes also express integrins and are potentially involved in CNS regeneration and development of neuroimmunological diseases, we focus on the role of astrocytes participating in the integrin signaling in the following sections.

## 2. Integrins and Regeneration Studies in Invertebrates and Fish

All multicellular animals express integrins that exist as heterodimers composed of one α and one β subunit. The fully sequenced genome of the fruit fly *Drosophila melanogaster* contains five α and two β integrin subunits. The nematode roundworm *Caenorhabditis elegans* has been shown to have two orthologues of α integrin (INA-2, PAT-2) and one ortholog of β integrin (PAT-3) [24]. Unicellular organisms also have integrins, a recent genetic analysis had shown that many of the key genes of integrin adhesion complex that had formerly been cited as crucial for metazoan origins have a much earlier origin [25].

On the basis of a phylogenetic reconstruction, Johnson et al. [24] divided vertebrate and invertebrate animal α integrins into six clusters, termed PS1 (vertebrate α3, 6, and 7), PS2 (vertebrate α5, IIb, V, and β), PS3, PS4 (vertebrate α4/9), collagen receptors (vertebrate α1, 2, 10, and 11), and leucocyte integrins of the immune system (vertebrate αE, L, M, D, and X). The PS3 group seems to be specific for insects. The PS4 group includes two mammalian α subunits, namely α4 and α9, can be considered to be a separate subgroup. These integrins recognize, in addition to ECM proteins, certain plasma proteins such as osteopontin, receptors belonging to the immunoglobulin superfamily, and vascular endothelial cell growth factors.

**Figure 1:**
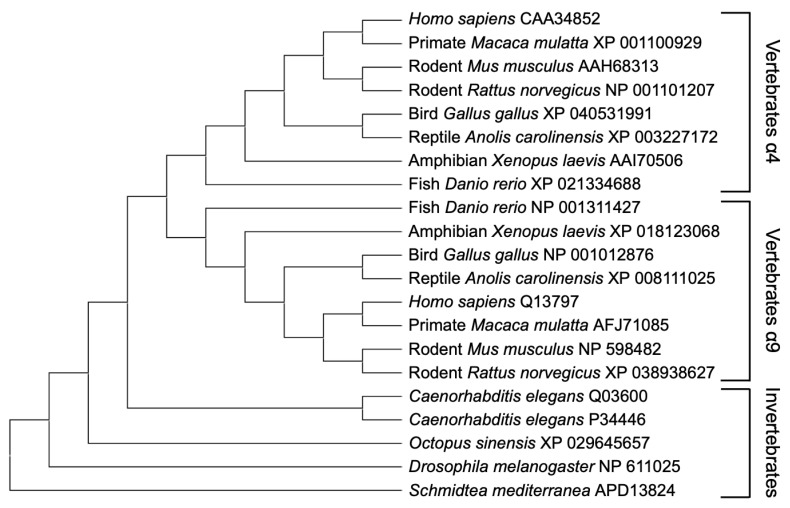
Phylogenetic relationship of PS4 integrin group. Protein sequences of the vertebrate integrins α4 and α9 and invertebrate integrins (α ina-1 and pat-2 of *Caenorhabditis elegans*, α4 of *Octopus sinensis*, αPS4 of *Drosophila melanogaster*, and α int-4 of *Schmidtea mediterranea*) obtained from the NCBI were aligned using the ClustalW algorithm, and a phylogenetic tree was drawn with Neighbor-Joining on MEGA11 [33].

We have researched TN-C and OPN [26,27,28,29,30,31], and have revealed that either of the molecules are indispensable for mammalian brain functions in concerted with α9 and β1 integrin as the receptors (see Section 4.4 and Section 4.5). Furthermore, recently, it has been reported that α9 integrin could be a therapeutic target for nerve injury [32]. Thus, we focused on α9 integrin and a subgroup member α4 integrin, and performed a molecular phylogenetic tree analysis for integrins of the PS4 cluster. We clearly showed that the vertebrate α4 subunit and α9 subunit, which are distinct from the invertebrate α4 subunit and α9 subunit, could have been evolutionary derived from the α4 subunit before the appearance of the first vertebrates (Figure 1). While integrin α9 is thought to be vertebrate-specific, integrins of the PS4 group (including Drosophila PS4) are independent from other PS groups and might have common functions in the CNS (see below).

Johnson et al. [24] divided the vertebrate and invertebrate animal β integrins into three clusters, namely, vertebrate A (β1, 2, and 7), vertebrate B (β3, 4, 5, 6, and 8), and invertebrate clusters.

### 2.1. Invertebrate (Planaria, Nematode, Fruit Fly, and Cephalopods)

Regeneration is a process that is ubiquitous among multicellular organisms. Some species of planarian flatworms (including the freshwater triclad, *Schmidtea mediterranea*) show autotomy, namely, when one adult flatworm is cut into two, it grows into two adults, and animals have adult regeneration ability for need to regulate, using specific signaling, cell proliferation, specification and patterning induced by injury. Bonar and Peterson identified that inhibition of one of four planarian integrin-alpha subunits, that is, α integrin 2, resulted in phenocopying of the regeneration of the normal brain architecture, but without normal cell-type regionalization, suggesting that a specific receptor controls brain organization through regeneration [34].

The nematode (roundworm) *Caenorhabditis elegans* is a well-known model for almost complete identification of the whole adult cell lineage and a fast life cycle. It has been reported that the TNS-1 (*C. elegans* homolog of vertebrate tensin) PTB domain is required for its association with the cytoplasmic region of the integrin β subunit PAT-3, and that this integrin pathway also promotes axonal regeneration through activation of the JNK cascade [35]. It has also been demonstrated that the integrin α subunit INA-1 activates the JNK pathway through the signaling complex CED-2–CED-5–CED-12, to promote axonal regeneration [36], and that the *C. elegans* integrin signaling network regulates axonal regeneration via the Src–RhoGEF–RhoA axis [37]. Single cell lineage analysis has also shown glia-derived neurons in adults whose progenitor cells are fully differentiated glia [38].

The fruit fly *Drosophila melanogaster* has also been shown to be an excellent model for genetic research. Drosophila neurons express integrins, heterodimeric cell surface receptors consisting of an α subunit and a β subunit. Neuron-specific overexpression of myospheroid (mys, a β integrin subunit) and multiple edematous (mew, an α integrin subunit) increase dendrite adhesion to the ECM [39].

Cephalopods (including the east Asian common octopus (*Octopus sinensis*)) are not only an attractive target for ethologic studies of animals with higher intelligence, but also a promising model for research in genomics [40]. Since cephalopods show regeneration of the arms, shell, and nerves [41,42], integrins of cephalopods may also contribute to these regenerations, as in vertebrates.

### 2.2. Vertebrates (Fish)

Many animals are used as models, but teleost fish are an excellent model for the study of regenerative systems of vertebrates and possess the ability to replace missing organs. These fish work as an ideal pathological model because of higher similarities with humans, neuroanatomical, neurochemical, and emotional/social behaviorally.

The CNS neurons of lower vertebrates, such as fish and amphibians, have been known to have the ability to regenerate after axotomy since Sperry’s work in the 1950s [43]. In contrast to the case in mammals and birds, neurons in the CNS of fish, such as the retinal ganglion cells, are capable of axonal regeneration and vision restoration. The axonal regeneration seems to be accomplished successfully in the fish visual pathway with some particular properties of the glial cells and neurons [44].

Mammalian neurons have been shown to express enhanced neurite outgrowth on salmon fibrin gel via the mediation of integrins [45]. Integrin of not only mammalian neurons, but also fish microglia and macrophages have been revealed to be involved in self optic nerve regeneration in fish [46,47]. Inhibition of tenascin C (TN-C) ameliorated axon regeneration and recovered the swimming behavior after spinal cord injury in adults, indicating that inhibitors of axonal regeneration are not completely lacking in the CNS environment of fish. Thus, similar to chondroitin sulfate proteoglycans (CSPGs), tenascin R may play a role in guidance during axonal regeneration. The lack of inhibitory molecules or cells and the presence of positive factors may induce CNS regeneration in fish. As potential therapeutics of CNS injuries, it may be valuable to consider some of these factors [48]. After traumatic brain injury, transformation of quiescent type I radial glial cells into proliferative type II radial glial cells similar to mammalian reactive astrocytes has been shown to be significantly suppressed in zebrafish harboring mutant glial maturation factor β [49].

A wide variety of fish species have been used for the study of regeneration, such as the lamprey, salmon, medaka, goldfish, and zebrafish (larva and adult), and have their pros and cons. Finally, we recommend one teleost fish, the Japanese eel (*Anguilla japonica*), known for its long spawning migration (about 3000 km) from Japan to the West Mariana Ridge (seamount chain) in the western North Pacific region [50]. They have also been used in the study of endocrinology because of their toughness to injury, and in behavioral experiments [51]. We developed an easy method for ablation of the telencephalon of the Japanese eel [52] and have observed their CNS regeneration and behavioral aspects of spatial learning and social behavior [53].

## 3. Integrins in Vertebrates (Mammals)

### 3.1. Integrin Signaling between Neurons and Astrocytes

Thy-1 is an abundantly expressed glycoprotein in neurons, which functions as an inhibitor of neurite outgrowth and acts as both a ligand, such as for integrins and other receptors, and as a receptor in Thy-1-expressing cells [54]. In the interaction between neuron and astrocytes, the αvβ3 integrin, as a receptor, directly binds to the Thy-1 present on neurons, stimulating the astrocytes [55]. Furthermore, αvβ3 integrin in astrocytes limits the neurites growth and also induces retraction of already existing processes by Thy-1 clustering induction [56]; it also triggers neurite retraction via the RhoA/ROCK pathway [57]. Moreover, upregulation of integrins in the astrocytes by inflammation induces neurite retraction by binding Thy-1, and formation of a ternary complex, i.e., Thy-1–αvβ3 integrin and Syndecan-4, alters astrocyte contractility and inhibits neurite outgrowth [58]. Astrocytic migration requires a large increase in the intracellular Ca^2+^ concentration and connexin 43 hemichannel opening, which are induced by Thy-1 via αvβ3 integrin in astrocytes [59,60]. Modulation of the astrocyte–neuron interaction in relation to this mechanism might contribute to axonal repair after CNS injury.

### 3.2. Role of Astroglial Integrins in the Functioning of the CNS

The largest integrin subfamily, the β1 integrin, is highly expressed in the mammalian neural stem cells (NSCs), and plays a role in the “niche” by regulating the growth factor responsiveness of NSC into either remaining as stem cells or differentiating and migrating away to shape the developing cortex [61]. Moreover, activation of integrin-linked kinase (ILK) in cultured adult NSCs from β1 integrin ablated mice reduced astrocytic differentiation, as β1 integrin restricted astrocytic differentiation of adult hippocampal NSCs mediated by ILK [62].

Selective ablation of αv or β8 integrin in astrocytes has revealed that αvβ8 integrin signaling links astrocytes to blood vessels and is essential for retinal angiogenesis [63]. In addition, β1 integrin is required for efficient blood vessel-guided migration of neuroblasts towards an infarct area and also their stable adhesion to laminin-rich astrocytes [64]. Thus, astroglial integrin signaling is indispensable for precise functioning of the CNS.

### 3.3. Integrins in Neurodegenerative Diseases

For neurons, integrin activation in neurite outgrowth and axonal regeneration have been extensively summarized [65]. Furthermore, integrin functions have, recently, been reviewed for effects of neuronal connectivity disruption in neurodevelopmental diseases, axonal outgrowth, synaptogenesis and synaptic maturation [17], and also for neuropsychiatric disorders [66].

Age-related macular degeneration (AMD) is characterized by a deficit in autophagy associated with deficiency of calcium and integrin binding protein 2 (CIB2), which are essential for visual functions [67]. Defects in the CIB2 protein have been reported to be related to deafness and/or vision deficits in humans, zebrafish, and drosophila [68].

Multiple sclerosis (MS) is a chronic auto-neuroinflammatory disease of the CNS driven by peripheral immune cells invading the target tissue to cause demyelination and neuronal death [69]. To overcome the BBB, the T cell α4β1 integrin interacts with endothelial vascular cell adhesion molecule 1 (V-CAM1) [70], and an antibody against α4β1 integrin (Natalizumab) has been commonly used in the treatment of MS [71]. The mechanism of T cell infiltration into the brain has not yet been elucidated, but recently, Zondler et al. reported that melanoma cell adhesion molecule (MCAM) signaling via phosphorylated PLCγ1 induced intracellular signaling leading to β1 integrin activation on human memory T cells, resulting in increased brain T cell infiltration [72].

### 3.4. Integrins in the Regeneration of the CNS

In adult mammals, CNS axons fail to regenerate, while PNS neurons support robust axonal regeneration over long distances. Petrova and Eva reported selective axonal transport of integrins as regeneration-associated receptors [13]. A key difference in the regeneration capability between the CNS and PNS is whether integrin is transport into the axons or not. Integrins can mediate PNS regeneration, but are excluded from CNS axons after development [73,74]. Thus, when integrins and carrier Rab11 endosomes in the CNS axons were manipulated in vitro, axonal regeneration was observed in the dorsal root ganglion (DRG), although the findings remain to be seen in vivo [75,76].

Expression of the integrin activator kindlin-1 in the DRG neurons only promoted limited regeneration in the spinal cord, whereas co-expression of α9 integrin with kindlin-1 induced extensive regeneration and functional recovery [77,78]. This latter strategy, however, failed in the CNS axons, because of the exclusion of integrins from the CNS axons due to changes in trafficking with the maturation of neurons [73].

The roles of integrins related to glycoproteins, such as laminin-, fibronectin-, and collagen-associated integrins, in axonal regeneration in PNS injury have been intensively examined, but no effective results for CNS injury have been obtained yet. A possible reason is that most axon guidance repulsive molecules embark inactivation of integrins [15]. The authors claim that modulation of integrins could be a general approach to promote axonal regeneration in the CNS. Collectively, in this review, we are going to focus on the integrin functions in relation to astrocytes and reactive astrocytes rather than neurons.

## 4. Integrin Involvement in the Roles of Astrocytes in Physiological and Pathological States

### 4.1. Integrins Involved in Astrocyte Functions

Neurons are generated from NSCs in the subventricular zone (SVZ) of the lateral ventricle in the adult mammalian brain [79,80]. In the SVZ, slow-dividing cells generate fast-dividing cells that migrate tangentially within astrocytic tubes along the rostral migratory stream (RMS) to the olfactory bulb. β1 integrins are required for the maintenance of these astrocytic tubes, and defects lead to ectopic migration of neuroblasts into the tissues around the RMS [81]. Thus, β1 integrin promotes cell–cell interactions that link neuroblasts to astrocytic chains.

*Clostridium botulinum* C3 transferase (C3bot) peptides enhance neuronal regenerative growth and connectivity, and seemed to show potential as treatment for spinal cord injury (SCI) [82,83]. C3bot harboring a mutated integrin-binding RGD (arginine-glycine-aspartate) motif (C3bot-G89I) showed reduced binding to even astrocytes releasing the intermediate filament protein vimentin, suggesting that vimentin and β1 integrin are binding partners and that their binding is involved in the internalization of C3bot into cultured astrocytes and astrocytes in SCI rats [84]. Thus, this mechanism of β1 integrin signaling initiated by C3bot with release of vimentin from astrocytes could be used as a therapeutic target for SCI treatment.

Prominent labeling for β1 integrin was observed in astrocyte processes near both unmyelinated nerve and myelinated nerve fibers in the normal optic nerve, while in a mouse model of glaucoma, in the presence of elevated intraocular pressure (IOP), apoptotic astrocytes were observed [85]. Thus, astrocytes normally exhibit junctions with the basement membrane, which are disrupted by IOP elevation. Membrane-bound β1 integrin is involved in mechano-sensitive signaling and is thought to be potentially beneficial or detrimental to axonal health.

Galectins, which are a family of β-galactoside-binding lectins consisting of 15 members in mammals, and their ligand glycoconjugates, including integrins, play diverse roles in both the physiology and pathology of the CNS [86]. In the adult mouse brain, galectin-1 binding activity has been detected on the β1 integrin-expressing cells in the subependymal zone (SEZ). Furthermore, β1 integrin has been shown to be required for galectin-1 functioning in neural progenitor cell (NPC) adhesion in vitro [87]. A genome-wide expression analysis, conducted to compare adult NSCs isolated from the SEZ and reactive astrocytes isolated 5 days after stab wound injury from the adult mouse cerebral cortex, revealed higher expression levels of galectin-1 and -3 levels in both types of cells, and addition of galectin-3 fully rescued the NSC phenotype in mutant animals [88,89]. Taken together, galectin-1 and -3 may contribute to providing a stem cell niche and play differential roles in neural generation via β1 integrin.

### 4.2. Integrin Signaling in Reactive Astrocytes

Astrocytes become reactive after brain/spinal cord injury or neuroinflammation involving the CNS, and reactive astrocytes show morphological changes and upregulation of some intermediate filament proteins such as GFAP, vimentin, and nestin, as well as of ECM molecules such as TN-C and chondroitin sulfate proteoglycans (CSPGs) [90,91,92,93]. It remains under debate as to whether this is a favorable reaction as an essential state for wound healing (good thing and beneficial), or an unfavorable reaction inhibiting axonal regrowth and neurite outgrowth (bad thing and detrimental) [94,95]. On the one hand, corroborating the notion that it is an unfavorable reaction, activated astrocytes form a glial scar which acts as a physical barrier to regenerating axons in the mammalian brain, and neurotoxic reactive astrocytes induce cell death in the CNS [96,97]. On the other hand, as evidence of its being a favorable reaction, a glial scar prevents the spread of neuronal death and inflammation around the wound site [98,99]. Moreover, Zamanian et al. termed A1 reactive astrocytes, which showed strong upregulation of various genes, as destructive to neurons (neurotoxic) and A2 reactive astrocytes, which release many neuroprotective neurotrophic factors [100]. A2 astrocytes show upregulated expressions of HMGB1 and β2 integrin, and promote adhesive interactions between the brain endothelium and endothelial progenitor cells [101,102].

Next, we focused on integrin signaling in reactive astrocytes to determine whether reactive astrocytes represent a favorable or unfavorable reaction to injury. Brain damage (i.e., traumatic brain injury, inflammation, or ischemia) is associated with upregulation of one of the astrocyte surface proteins, i.e., integrins, and proinflammatory cytokines and ECM proteins are released to generate a stiffer matrix, which leads to the formation of a glial scar. It is known that interaction between upregulated type I collagen and its receptor β1 integrin and N-cadherin-mediated cell adhesion play major functions around the lesion site of a SCI [103]. Furthermore, administration of anti-β1 integrin antibody during the scar formation period has been found to inhibit the interaction between type 1 collagen and Ras, and prevented transformation of reactive astrocytes to scar-forming astrocytes, thereby, preventing scar formation, promoting axonal regrowth, and improving functional recovery in the injured spinal cord [104]. They showed that normal migration of reactive astrocytes to limit the compact lesion area induced functional recovery, whereas their impaired migration resulted in a wide lesion area and poor functional recovery after the SCI. During the scar-forming phase, type I collagen is highly expressed in the injured spinal cord and induces astrocytic scar formation via the integrin-N-cadherin pathway [105].

Astrocyte adhesion and contractility are induced by αvβ3 integrin/Thy-1 signaling, and are enhanced and accelerated by mechanical stress, as demonstrated using a magnetic bead assay [106]. The mechanoreceptor αvβ3 integrin could be a new target for the negative effects of reactive astrocytes in an injured brain. In accordance with these data, astrocytes are responsive as mechanosensitive cells via the mediation of integrins.

In an injured brain, expression of the actin-binding protein drebrin (DBN) is upregulated in reactive astrocytes, and is essential for glial scar formation and maintenance in vivo. DBN-mediated membrane trafficking via β1 integrin, in inducing reactivity of astrocytes, has been shown to be an important neuroprotective mechanism following traumatic brain injury in mice [107]. Type I collagen is highly expressed in the spinal cord during astrocytic scar formation phase via the integrin-N-cadherin pathway, and its blockade prevents glial scar formation, resulting in improved axonal regrowth [105]. Therefore, β1 integrin could be effectively targeted to promote SCI recovery.

**Figure 2:**
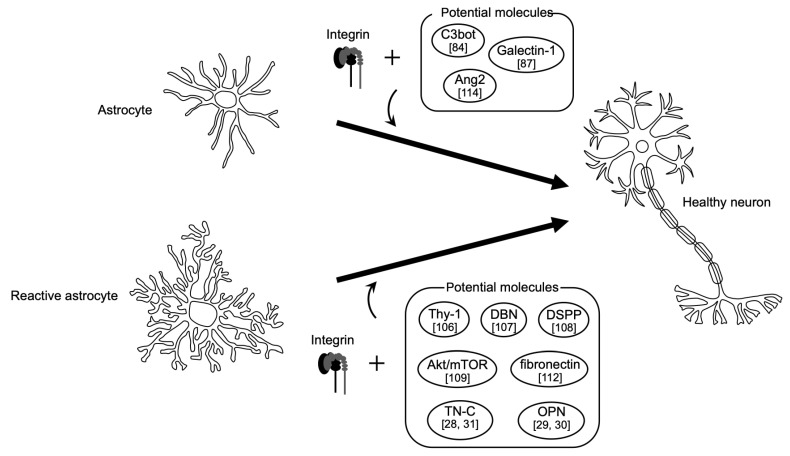
Involvement of integrins in CNS regeneration in collaboration with potential factors. Modulation of integrins with potential factor(s) stated in the main text, and possibly also the environment, could convert astrocytes or reactive astrocytes to healthy neurons for induction of neuro-regeneration in the mammalian CNS. The number(s) in square brackets beside the factor names are the reference numbers cited in the main text [28,29,30,31,84,87,106,107,108,109,112,114].

It has been reported that dentin sialophosphoprotein (DSPP), a member of the small integrin-binding ligand N-linked glycoprotein family, interacts with β6 integrin to promote cell proliferation, differentiation, and migration [108]. In fact, overexpression of DSPP increased activation of the Akt/mTOR pathway in astrocytes, which might promote proliferation and migration of reactive astrocytes [109]. Thus, targeting DSPP appears to be an effective therapeutic strategy for treating CNS injury related to glial scar formation. As Adams et al. advocate [94], we propose to target the coordination of reactive astrocytes and integrin signaling for developing therapies for neurodegenerative diseases (Figure 2).

### 4.3. Integrins in Neuroinflammation Mediated by Astrocytes

Astrocytes interact not only with neurons, but also with microglial cells, oligodendrocytes, endothelial cells, and peripheral immune cells in the event of neuroinflammation [110,111]. Yoshizaki et al. revealed that after chronic spinal cord injury in mice, microglial inflammation was enhanced by reactive astrocytes via the fibronectin/β1 integrin pathway. They showed that intralesional administration of anti-β1 integrin antibody in the subacute phase of SCI suppressed the production and secretion of tumor necrosis factor α, an inflammatory cytokine, and glial scar formation by switching of the microglial phenotype to an anti-inflammatory phenotype [112]. Targeting β1 integrin signaling as a potential therapy for SCI could have a potential risk due to the fact that it could form a lot of potential heterodimers, and that might induce integrin crosstalk.

It has been reported that anti-β1 integrin antibody exerts therapeutic effect by preventing glial scar formation [105]. Furthermore, the antibody also effectively blocks the interactions between reactive astrocytes and microglial cells via fibronectin. From these data, modulation of integrin signaling could be attempted for developing treatments against neuroimmunological diseases.

Another inhibitor, ATN-161, of α5β1 integrin, has been reported to reduce the infarct volume, edema, and infiltration of immune cells into the brain and functional deficit, and to be neuroprotective in MCAO mice, a mouse model of ischemic stroke [113].

In diabetic retinopathy, angiopoietin (Ang2) induces astrocyte apoptosis in the presence of high glucose via the αvβ5 integrin/GSK3β/β-catenin pathway [114]. Thus, Ang2/integrin signaling could be targeted for inhibiting vascular leakage by astrocyte loss in early diabetic retinopathy.

Astrocytes are also known to be involved in the pathogenesis of Alzheimer’s disease (AD). Functional changes, such as disrupt gliotransmission, neurotransmitter uptake, and altered calcium signaling in astrocytes have been observed in the brains of individuals with AD in the presence of amyloid beta. Actually, downregulation of astrocytic cytoskeletal proteins, such as actin β, dynein, and α integrin, is observed in human AD patients [115]. Astrocyte αv/β1 integrin receptor binding to recombinant human Tau, which mediates the entry of Tau fibrils into astrocytes, and induction of integrin signaling leads NFκB activation, causing upregulation of proinflammatory cytokines and chemokines, induction of a subgroup of neurotoxic astrocyte markers, and release of neurotoxic factors in primary cultures of astrocytes [116]. Since ablation of reactive astrocytes has been shown to exacerbate memory deficits in a mouse model of AD [117], manipulation of astrocytes could be useful for clarifying the mechanisms of memory loss in such conditions as tauopathies.

Glial scar, formed by activated astrocytes, have been thought to interfere with CNS regeneration. Anderson et al. showed that preventing astrocyte scar formation significantly reduced axonal regrowth, and their RNA sequencing revealed that astrocytes at the site of SCI expressed multiple axon-growth supporting molecules [118]. Furthermore, the transcriptome profile has been reported of neuroinflammatory astrocyte subtypes induced by bacterial cell wall endotoxin lipopolysaccharide (LPS) injection in mice [119]. Since astrocytes and reactive astrocytes have been implicated in traumatic SCI [120], targeting of glial scar formation after a SCI might be helpful to induce CNS regeneration.

### 4.4. TN-C Function in Astrocytes via Integrins

The authors have focused on TN-C and osteopontin (OPN), which are ligands for the integrin family of receptors in the brain, to investigate the neuroinflammatory functions of astrocytes. For example, TN-C has functional roles such as the BBB recovery from breakdown caused by a brain injury, proliferation of astrocytes, and balancing between neuroprotective- and neurotoxic-cytokine production, which were examined after a stab wound injury to mice cerebral cortex and in primary culture of astrocytes [26,27,28]. First, we summarize the roles that TN-C plays in integrin signaling involved in the functioning of astrocytes.

TN-C is one of the ECM glycoproteins of the CNS that is involved in CNS development, and its expression is upregulated after brain or spinal cord injury [121,122]. TN-C is also known to have immunomodulatory roles in neurodegenerative disorders, and to be highly associated with astrocyte activation and glial scar formation [123].

Neurons transfected with α9 integrin and kindlin-1 in vitro grew axons for long distances on TN-C, however, a much lower degree of regeneration was observed in vivo [124]. They hypothesized that major inhibitory molecules, not present in the in vitro culture system, in the adult CNS might inactivate integrins, preventing them from binding with TN-C. Actually, Tan et al. revealed using adeno-associated virus vector expressing α9 integrin in the adult rat DRG neurons, a number of axons growing into the dorsal root entry zone in vivo. However, the inhibitory substrates CSPG and Nogo blocked the growth by inactivating integrins [77]. Since expression of the α9 integrin subunit was downregulated in the mature corticospinal tract (CST), grafting of lentivirus-mediated α9 integrin-overexpressing induced pluripotent stem cell (iPSC)-derived human neural progenitor cells (hNPCs) resulted in increased neurite outgrowth in the presence of TN-C in the developing CST [125]. Using a scratch wound in the primary culture of astrocytes, it was shown that extracellular vesicle functions and wound recovery are mediated by β1 integrin related to IL-1β treated TN-C-bound-primary culture [126]. Although TN-C exerts an inhibitory effect on mature axons, when specifically bound to α9β1 integrin, TN-C can promote neurite outgrowth and axonal regeneration [127].

Recently, we reported TN-C as an inhibitor of neuronal death after stab wound injury in mouse brain and in a primary culture with LPS in the presence of 2-carba-cyclic phosphatidic acid (2ccPA) [31]. In this study, TN-C secreted from astrocytes had a neuroprotective role, but only in the presence of 2ccPA. Thus, although TN-C and integrins are becoming well-known as being neuroprotective, additional factor(s) and conditions seem to be necessary for their inducing neuronal regeneration in vivo.

### 4.5. OPN Functions in Astrocytes Mediated via Integrins

Next, we summarize the functions of OPN mediated via integrins playing a role in astrocyte functions.

OPN is a proinflammatory cytokine inducer and a pleiotropic protein involved in a wide range of cell functions, such as bone development and remodeling, vascularization, wound healing, immune response, and inflammation, and also tumor metastasis [128,129]. OPN is weakly expressed in the brain under normal conditions, while it is strongly expressed in the microglia and astrocytes, exerting a neuroprotective effect after brain injury [130].

Recently, we demonstrated that in the injured cerebral cortex after a stab wound and in a primary culture of astrocytes with LPS, an inflammation inducer, reactive astrocytes also expressed OPN, and that OPN exerted its neuroprotective role both in vivo and in vitro via the mediation of α9β1 integrin [29,30]. Although OPN utilizes various combinations of integrin heterodimers, binding through the RGD domain, the precise receptors it binds to depends on the pathological conditions. Actually, several cell surface integrins, such as αvβ1, αvβ3, αvβ5, α4β1, α8β3, and α9β1, and also CD44 variants are known to serve as receptors for OPN [131,132].

OPN has been reported as a potential cerebrospinal fluid biomarker in amyotrophic lateral sclerosis [133,134] and also in neuromyelitis optica (NMO), an inflammatory disease of the CNS affecting the optic nerve and spinal cord [135]. It has been shown that OPN in the cerebrospinal fluid in NMO patients bound with integrin αvβ3 to promote macrophage chemotaxis by activating the phosphoinositide 3-kinase and MEK1/2 signaling pathways.

The RGD domain of OPN interacts with the integrins αvβ3 and β1, and seems to play a role in the neuroprotective actions of OPN in models of Parkinson’s disease. Broom et al. reported that both OPN and the synthetic 15-mer RGD domain of OPN were equally protective of increased glial-derived neurotrophic factor and brain-derived neurotrophic factor levels against 1-methyl-4-phenylpyridinium exposure of ventral mesencephalic cultures, accompanied by a decrease in the numbers of activated microglia, but no change in the astrocyte number [136]. This finding suggests that other domains of OPN may also be needed for their neuroprotective role.

Human immunodeficiency virus does not enter or replicate in neurons, but can infect microglia and astrocytes to induce neurotoxicity and neuroinflammation. Zhu et al. showed that extracellular OPN blocked the negative effects of the envelope on neurite growth via the integrin mTORC1/2 signaling pathway, which is involved in long-term potentiation and long-term depression [137], learning and memory, neuronal survival, differentiation, and morphogenesis [138].

OPN signaling through β1 integrin is not only required for stimulation and maintenance of neurite growth in cultured primary rat cortical neurons, but also to protect neurons from excitotoxicity [139]. Thus, β1 integrin is the key factor involved in higher brain functions mediated by OPN.

### 4.6. Integrins Functions in the BBB

The BBB, a picket line, constitutes a neurovascular unit formed by microvascular endothelial cells (EC), pericytes, and astrocytes that prevent certain molecules from passing from the blood into the brain. Astrocyte endfeet and pericytes that contact the cerebrovascular endothelium, and specialized connections with the tight junction (TJ), i.e., claudin-5, between the EC, and zonula occludens 1 (ZO-1) and occludin are involved in the integrity of the ECM of the basal lamina [140,141].

Integrins contribute significantly to angiogenesis and vascular remodeling mediated by thrombospondin-1/integrin/YAP signaling [142,143]. The role of integrins and ECM functions in the endothelial cells of the BBB in stroke or EAE have already been reported in previous papers [144,145,146]. In the present paper, we mainly focus on pericyte and astrocyte functions in the BBB mediated via integrin signaling.

Loss of the tight connections of astrocytes to endothelial cells and reduced expressions of pericyte markers and of various integrins such as α6, α3β1, and β1 with loss of TJ integrity have been associated with enhanced permeability of the BBB in mice [147]. Nakamura et al. showed, in an ischemic stroke mouse model, that perlecan, a major heparan sulfate proteoglycan of the basement membrane, regulated the pericyte dynamics through α5β1 integrin in the maintenance and repair of the BBB [148]. These data suggest that β1 integrin is involved in the integrity of the BBB.

ECM epidermal growth factor-like protein 7 (EGFL7) secreted by the ECM is increased in patients with MS and in EAE mouse model of MS. T lymphocytes expressing αvβ3 integrin adhered to EGFL7. Recombinant EGFL7 has been shown to improve EAE, tighten the BBB, and reduce neuroinflammation in mouse [149]. Thus, EGFL7/αvβ3 integrin signaling could serve as a new therapeutic target for MS.

Chronic mild hypoxia (CMH) promoted a strong vascular remodeling response in the spinal cord blood vessels via an α5β1 integrin-mediated mechanism in mice [150]. CMH triggered astrocyte activation specifically in the gray matter and the newly generated vessels grew towards neuron-rich areas and were also closely associated with astrocytic endfeet.

## 5. Conclusions

Integrin signaling mediates a variety of functions, not only in neurons but also in astrocytes in the brains of mammals, fish, as well as invertebrates. In this review, we have summarized the roles of integrin signaling in the functioning of astrocytes and reactive astrocytes, which could provide further insight for unlocking the potential for valuable neuronal regeneration in CNS injury and disease in humans.

## Abbreviations

BBB	blood brain barrier
CNS	central nervous system
CSPG	chondroitin sulfate proteoglycan
DRG	dorsal root ganglion
ECM	extracellular cellular matrix
GFAP	glial fibrillary acidic protein
LPS	lipopolysaccharide
NSCs	neural stem cells
OPN	osteopontin
PNS	peripheral nervous system
RGD	Arg-Gly-Asp
SCI	spinal cord injury
SVZ	subventricular zone
TJ	tight junction
TN-C	tenascin C
